# Dynamic Tensile Behaviors of HRB500E Connected with Extrusion Sleeves at Different Strain Rates

**DOI:** 10.3390/ma16020828

**Published:** 2023-01-14

**Authors:** Wanxu Zhu, Dongwen Wu, Yiling Chen, Yongqi Su, Shiyuan Liang

**Affiliations:** 1 Guangxi Engineering Research Center of Intelligent Structural Material, Guilin University of Technology, Guilin 541004, China; 2 Guangxi Key Laboratory of Geotechnical Mechanics and Engineering, Guilin University of Technology, Guilin 541004, China; 3 Liuzhou OVM Machinery Co., Ltd., Liuzhou 545005, China; 4 Guangxi HCM Technology Co., Ltd., Guilin 541004, China

**Keywords:** dynamic, nuclear containment, strain rate, extrusion sleeve, HRB500E

## Abstract

In this paper, the connection performance of extrusion sleeves and the strain rate effect on 500 MPa-grade hot-rolled ribbed bar(HRB500E) connected with extrusion sleeves under a range of testing strain rates from 1.079/sto1.395/s, similar to what would be caused by an impact, were explored. The test results showed that, under strain rates likely caused by aircraft impact, the specimens mostly failed due to breaking outside the joint length. Furthermore, there was no relative slip between the rebar and the extrusion sleeve, indicating that the connection was stable and reliable in the used experimental parameter field. The percentage total elongation at maximum force (*Agt*) of HRB500E spliced by the extrusion sleeve showed an exponential decline with the increase in the strain rate, indicating a clear strain-rate sensitivity. The average deviation between the dynamic increase factors (*DIF*) calculated using the modified Cowper–Symonds formulas and the experimental values was within 5.4%, which can better reflect the strain rate effect on the strength of the spliced connection. The *DIFy* of sleeve-spliced rebars was higher than that of unspliced rebars, and the ratio of the *DIFy* of sleeve-spliced rebars to the *DIFy* of unspliced rebars increased with the strain rate. The experimental results can provide a basis for an optimized design of the aircraft impact-resistant extrusion sleeve rebar connections.

## 1. Introduction

In recent years, the risks caused by impacts to nuclear power plants, such as nuclear leakage, have attracted considerable worldwide attention, and the impact resistance requirements of nuclear power plant containment structures have become increasingly stricter. Dynamic loads, including impact, must be considered in the design of major national defense projects, such as nuclear power plants. Previous studies [1,2,3,4,5,6,7,8,9] showed that the strain rate of stressed rebars in reinforced concrete structures under impact may exceed 1.0/s; however, the connection performance and mechanical behavior of reinforcement joints under impact conditions are not clear yet. Therefore, more research on this specific problem is necessary.

The impact resistance of the third-generation nuclear power reactors, including China′s Hualong One, is regarded as the main performance characteristic. At the same time, a large number of studies have been conducted by various countries to evaluate the ability of the third-generation nuclear power reactors to resist the deliberate impact. The Nuclear Regulatory Commission (NRC) of America amended federal regulation 10 CFR 50 in 2009 to require applications for new nuclear power plants to assess the impact of a large commercial aircraft strike on the plant [10]. In 2016, the National Nuclear Safety Administration of China issued a new version of Nuclear Power Plant Design Safety Regulations (HAF 102—2016) [11], which also put forward requirements to combat the impact of large commercial aircraft. As the main force material of the concrete structure of nuclear power containment, the reinforcement and its joints are inevitably affected by the dynamic load. Numerous studies on the strain rate effect in rebars have been carried out. Malvar [12] et al. conducted experimental dynamic studies on the rebars with a yield strength of 290–710 MPa and proposed dynamic increase factor formulas for the rebar yield strength and ultimate tensile strength (UTS) based on the test results. Yang [13] et al. conducted a quasi-static tensile test and a dynamic tensile test on Q550 rebars under different strain rates. They found that the yield strength of Q550 rebars increased with the strain rate, but the strain-rate sensitivity of Q550 rebars was lower than that of ordinary low-carbon steel rebars. Zeng [14] et al. carried out dynamic tensile tests on HRB500E rebars, modified the Cowper–Symonds and the Malvar formulas for predicting dynamic yield stress based on the test results, and verified the Johnson–Cook formula and its modification. Lin [15] et al. studied the mechanical properties of HRB500E rebars under a strain rate of 4.9~59.0/s and established dynamic constitutive models for two types of HRB500E rebars based on the test results. Qiao [16] et al. studied HRB600 rebars under a strain rate of 0.0000395~0.0827/s and found that the strain-rate sensitivity of HRB600 rebars was lower than that of low-strength rebars. Wang [17] et al. studied the dynamic mechanical properties of HRB400E and HRB500E rebars and proposed a dynamic constitutive model suitable for the specimens used in the test. As can be seen from this literature survey, a broad consensus on the strain rate effect in rebars has been reached in the academic community.

However, only limited studies on the strain rate effect on rebar joints have been conducted in recent decades. Given that the strain rate effect on rebars will improve their strength, the design of rebar joints based on the original design criteria may cause rebar joints to fail before the failure of the rebars themselves. Preliminary studies on the connection performance of rebar joints under dynamic impact were conducted by some researchers. To highlight the dynamic characteristics of the fully grouted sleeve connection at loading rates of 0.6 mm/s, 6 mm/s, and 60 mm/s, Yin [18] et al. conducted static and low-speed dynamic tensile impact tests on 29 specimens and proposed that the connection failure of fully grouted sleeves was fundamentally determined by bond strength. To study the effect of impact load on rebar joints, Hu [19] et al. elaborated the principle of the impact-resistant tensile test and carried out process evaluation and improvement on the aircraft impact-resistant mechanical rebar splice of the Hualong One reactor in China. Feng [20] et al. conducted a sensitivity analysis on APC shells to determine the sensitive area of wall impact, thus narrowing the application scope of special mechanical splices in the design of anti-plane crash (APC) shells. Rowell [21] et al. compared the performance of different types of mechanical splices tested under the same strain rate and compared them to the requirements of UFC3-340-02. However, due to the lack of equipment to perform the impact test, most of the existing equipment cannot complete the rapid tensile of large tonnage specimens, resulting in a lack of domestic and foreign research on the dynamic mechanical properties of large diameter high-strength steel bars and the connection properties of connectors under impact load and a lack of relevant research results.

Therefore, in order to obtain the dynamic mechanical properties of high-strength rebar and the connection properties of steel bar extrusion sleeves for anti-plane crash nuclear containment under impact conditions, the impact test of anti-impact joints adapted to high-strength steel HRB500E at five strain rates of 1.395/s, 1.348/s, 1.298/s, 1.184/s and 1.079/s was carried out.

## 2. Theory of Strain Rate Effect in Rebars

### Formulas for Dynamic Increase Factor of Rebar Strength

The standard yield strength and ultimate strength of rebars are two important mechanical indices for rebar joints’ design according to JGJ/T163-2013 [22], see Table 1. The strain rate effect of the sleeve-spliced rebars’ strength subjected to impact loading can be determined to provide the basis for the design of the sleeves used in such applications as nuclear power plants. The strain rate effect on the strength of rebars can be quantified with the dynamic increase factor (DIF). The dynamic increase factor of yield strength (*DIF_y_*) and the dynamic increase factor of ultimate strength (*DIF_u_*) represent the ratios of the corresponding dynamic stresses to the quasi-static stresses. The existing calculation methods for the DIF of the strength of rebars include the Cowper–Symonds formulas [23] and the Malvar formulas [12].

The Cowper–Symonds formula for *DIF_y_* is as follows:

(1) $$DIF_{y}=\frac{f_{dy}}{f_{sy}}=1+{\left(\frac{\dot{\varepsilon }}{D_{1}}\right)}^{\frac{1}{q_{1}}}$$

where *f_dy_* represents the dynamic yield strength, *f_sy_* is the quasi-static tensile yield strength, and *D*_1_ and *q*_1_ are formula coefficients.

The Cowper–Symonds formula for *DIF_u_* is as follows:

(2) $$DIF_{u}=\frac{f_{du}}{f_{su}}=1+{\left(\frac{\dot{\varepsilon }}{D_{2}}\right)}^{\frac{1}{q_{2}}}$$

where *f_du_* represents the dynamic ultimate strength, *f_su_* is the quasi-static ultimate strength, and *D*_2_ and *q*_2_ are formula coefficients.

## 3. Experimental Program

### 3.1. Test Object

**Figure 1:**
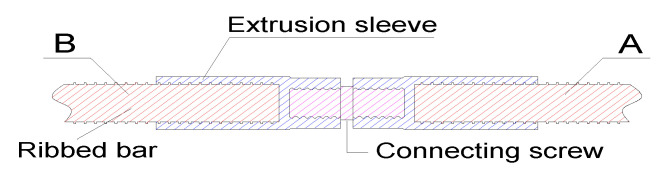
Sectional view of reinforcement mechanical joint specimen. “A” is the tension end and “B” is the fixed end.

A novel type of extrusion sleeve was investigated through dynamic tensile impact tests. The extrusion sleeve comprises two sleeves and connecting screws, see Figure 1. The two spliced rebars, A and B, are placed in the sleeves, which are then squeezed. First, rebar A is installed in the sleeve, and then a screw is tightened. After that, the sleeve of rebar B is screwed onto the other end of the connecting screw. The extrusion sleeves and the connecting screws were made of 40Cr alloy steel with a yield strength of at least 785 MPa and a tensile strength of at least 980 MPa. The measured quasi-static mechanical properties of the HRB500E high-strength rebars are shown in Table 2.

When the tensile test is carried out at high speed, the tensile test is completed in an instant. Thus, it can be considered that the deformation of the specimen changed uniformly with time during the tensile test. The strain rate can be determined by the following formula from GB/T 30069.2-2016 [24]:

(3) $$\varepsilon =\frac{\Delta L}{L-L_{0}}$$

(4) ΔL=υ×T

(5) $$\overset{\cdot }{\varepsilon }=\frac{\varepsilon }{T}=\frac{\upsilon \cdot T}{(L-L_{0})\cdot T}=\frac{\upsilon }{L-L_{0}}$$

where υ represents the loading speed, *L* stands for the total length of the specimen (1000 mm in this experiment), $\dot{\varepsilon }$ refers to the strain rate, and *L_0_* is the sleeve length.

According to Equation (5), the strain rate of the specimen varies with changes in loading speed. The dynamic tensile impact tests of the HRB500E rebars spliced using the novel extrusion sleeve were conducted at five different strain rates, namely, 1.395/s, 1.348/s, 1.298/s, 1.184/s and 1.079/s, through the control variable method. There were three specimens tested at each strain rate, i.e., 15 specimens in total.

The length of all specimens was controlled within 1250 ± 5 mm, and the clamping length at both ends of the specimen was 250 mm, i.e., the effective length, *L*, of the specimens was 1000 mm. The specimen specifications are listed in Table 3.

### 3.2. Testing Machine

**Figure 2:**
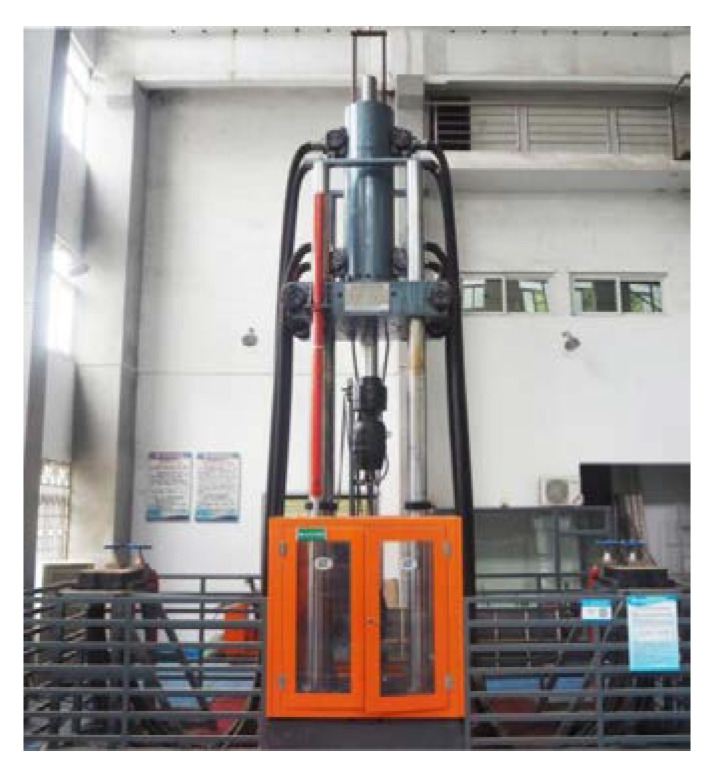
Electric hydraulic tensile impact test machine.

**Figure 3:**
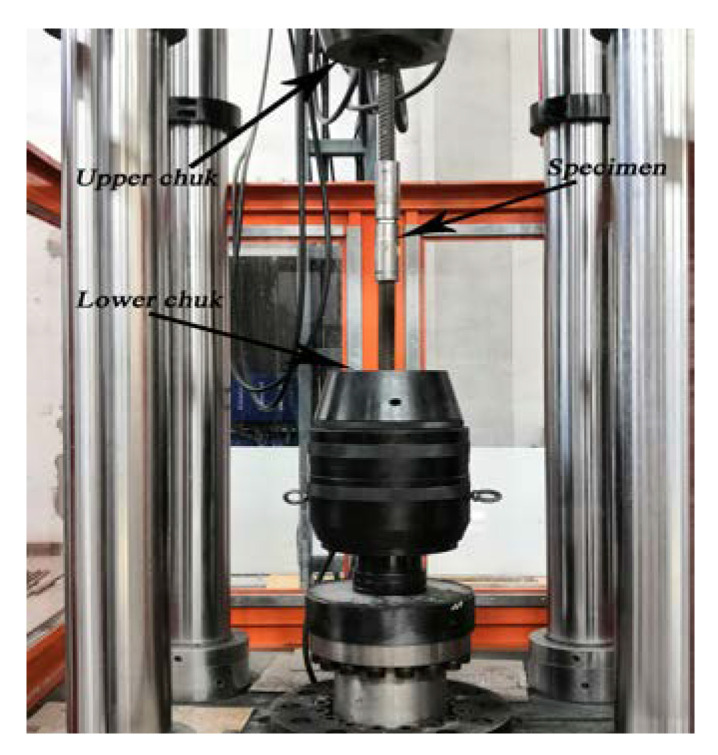
Specimen installation.

An electric-hydraulic tensile impact test machine was used for testing (Figure 2), which can provide a maximum impact force of 2000 kN and a maximum impact speed of 1500 mm/s. This machine is the latest specially developed high-stiffness tensile impact test machine designed for dynamic loading, which satisfies the special requirements for testing the seismic performance of rebars and their joints used in construction engineering. This test machine can be used for conventional static mechanical property tests and tensile impact tests. Furthermore, the mechanical property tests, such as the fatigue life of various materials and parts, can be carried out after the upgrade of the pump system. The test machine employs a vertical four-pillar structure, which facilitates the accurate alignment of the test specimens and reduces the flexural-torsional deformations. In addition, if required, the height of the test space can be adjusted up to 3200 mm by changing the position of the movable crossbeam. The upper and lower hydraulic chucks independently clamp and control the rebar joint specimen during testing to prevent the broken specimen from flying out and ensure the safety of experimenters. The sampling frequency of the measurement and control system was 1000 Hz (i.e., the sampling period was 1.0 ms). A specimen installed in the testing machine is shown in Figure 3.

## 4. Results and Discussion

### 4.1. Specimen Failure Modes

**Figure 4:**
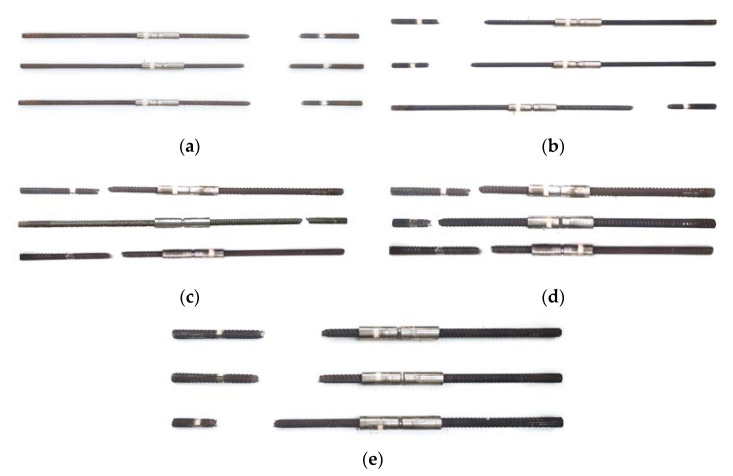
The failure mode of the specimen after the dynamic tensile test. (**a**) Φ16; (**b**) Φ20; (**c**) Φ25; (**d**) Φ32; (**e**) Φ40.

Figure 4 demonstrates the specimen failure modes. There were three common mechanical splice failure types: rebar fracture failure, joint tensile fracture failure, and rebar pull-out. The dynamic tensile impact test for rebar joints requires that all specimens break outside the joint length (sleeve length plus twice the diameter of rebar at the sleeve left and right ends). The experimental results for other specimen groups are displayed in Table 4. From Figure 4 and Table 4, it can be seen that all specimens satisfied the requirement that the rebar joints be broken beyond the joint length in the dynamic tensile impact test. For example, the average distance from the fracture to the joint in the Ø16 specimen group was 282 mm, i.e., it was greater than 32 mm. The extrusion sleeves caused no damage to the rebars, and the relative slip between the extrusion sleeves and the rebars was very small, demonstrating that the rebar joints were stable and reliable within the strain rate range of the impact.

### 4.2. Impact Deformation Performance of HRB500E Spliced with Sleeves

In the quasi-static uniaxial tensile condition, the maximum elongation of rebars spliced by sleeves generally reached 5%, but it tended to decrease under the instantaneous tensile impact. Table 5 reveals that the third-generation nuclear reactors in China, such as the Hualong One reactor, require that the relative total elongation at maximum force, *A_gt_*, of the mechanical splice be higher than 5%, and the French EPR nuclear reactor requires that *A_gt_* of a mechanical splice under the dynamic tensile impact be greater than 7.5%. In this study, *A_gt_* was calculated based on ENISO 15630-1 as follows [25]:

(6) $$A_{gt}=A_{g}+\frac{\sigma_{\max}}{2000}$$

(7) $$\sigma_{\max}=\frac{F_{\max}}{A_{N}}$$

where *A_g_* represents the relative non-proportional elongation at failure, *σ*_max_ refers to the maximum stress in the tested rebar, and *F*_max_ stands for the maximum axial force of the specimen in the instantaneous tensile test. The average nominal cross-sectional area, *A_N_*, is determined by dividing the weight of the reference rebar by the mass density and rebar length.

The dynamic mechanical performance parameters of specimens at different strain rates are shown in Table 6, where it can be seen that *A_gt_* of all specimens was higher than 5%. According to the design requirements for the French EPR nuclear reactors and the Hualong One reactor, as well as those stipulated in JGJ107, all specimens conformed to the technical design requirements of the Hualong One reactor. To be specific, all specimens met the technical requirements of the EPR nuclear reactors for the rebar mechanical splice joints (i.e., *A_gt_* was greater than 7.5%), except for the specimens tested at the strain rate of 1.381/s, for which *A_gt_* was less than 7.5%.

**Table 5:** Technical requirements comparison.

Technical Documents	Percentage Non-Proportionnal Elongation at Failure	Percentage Total Elongation at Maximum Force
GJ107-2010 Class I	*A_sgt_* ≥ 6.0%	No involving
EPR reactor	*A_sgt_* ≥ 7.5%	*A_gt_* ≥ 7.5%
HPR 1000	*A_sgt_* ≥ 6.0%	*A_gt_* ≥ 5.0%

**Figure 5:**
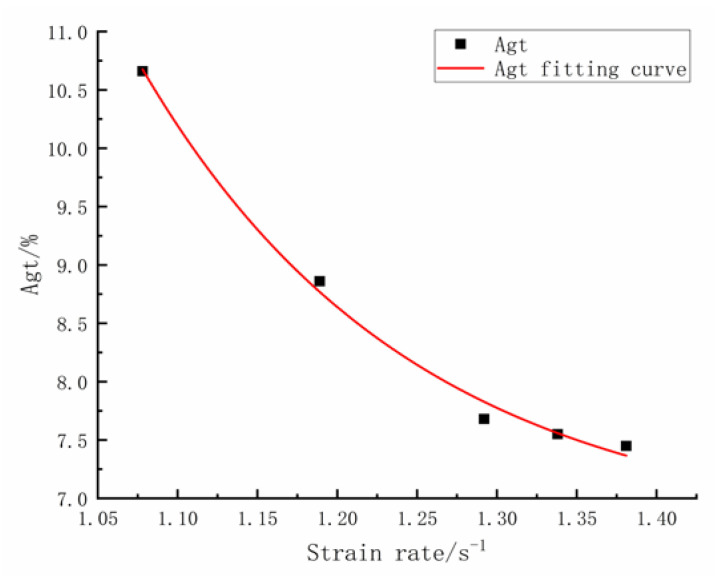
The change trend of strain rate—*A_gt_*.

Figure 5 shows an analytical curve fitted to the *A_gt_* and strain rate data, which was as follows:

(8) $$A_{gt}=6.69+2242.94e^{\frac{\overset{\cdot }{\varepsilon }}{0.172}}\;R^{2}=0.989$$

The analytical formula agrees well with the test results, giving a squared correlation coefficient of 0.989. From Figure 5 and Equation (8), it can be concluded that *A_gt_* of HRB500E anti-seismic rebars spliced with extrusion sleeves exponentially decreased with the increase in strain rate, displaying clear strain-rate sensitivity, while it essentially satisfied the technical requirements of the third-generation nuclear reactors, such as the Hualong One reactor.

**Table 6:** Mechanical indexes of specimens under different strain rates.

Test Group	Strain Rate//s	Yield Strength/MPa	Ultimate Tensile Strength/MPa	*A_g_*/%	*A_gt_*/%
Φ16	1.395	656.41	847.56	7.03	7.45
Φ20	1.348	655.62	849.74	7.13	7.55
Φ25	1.298	649.22	851.11	7.26	7.68
Φ32	1.189	647.96	858.1	8.43	8.86
Φ40	1.079	635.34	859.33	10.23	10.66

### 4.3. Strain Rate Effect on Strength

The Cowper–Symonds formulas for the traditional rebars without sleeve splices were modified, and the test data were used to accurately calculate the dynamic yield strength and dynamic ultimate tensile strength of HRB500E rebars spliced with sleeves in the strain rate range of the impact.

**Figure 6:**
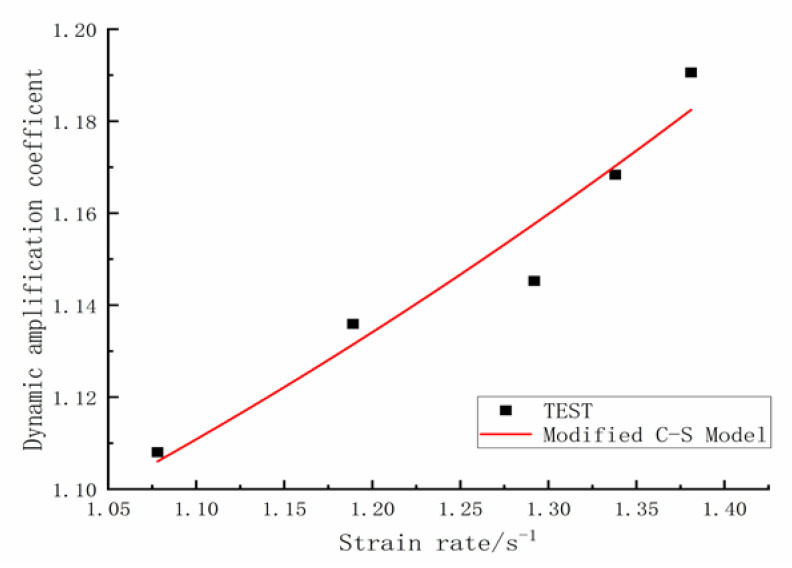
Cowper–Symonds model fitting curve of DIFy.

Figure 6 shows the results of fitting the *DIF_y_* Cowper–Symonds formula to the data. Parameter *D*_1_ equals 3.0, *q*_1_ equals 0.456 and the squared correlation coefficient reaches 0.917. Thus, the fitted formula results are in good agreement with the test results, which demonstrates that the Cowper–Symonds formula accurately reflects the strain rate effect on the yield strength of rebars spliced with sleeves.

**Figure 7:**
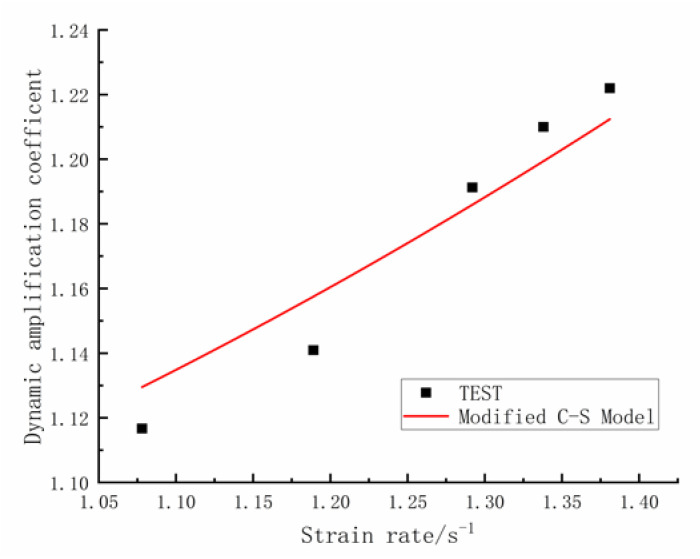
Cowper–Symonds model fitting curve of *DIF_u_*.

Figure 7 shows the results of fitting the *DIF_u_* Cowper–Symonds formula to the data. Parameter *D*_2_ equals 3.0, *q*_2_ equals 0.5 and the squared correlation coefficient reaches 0.918. The fitted formula results are again in good agreement with the test results, which shows that the modified Cowper–Symonds formula correctly reflects the strain rate effect on the ultimate strength of rebars spliced with sleeves.

The deviations between the fitted *DIF_y_* and *DIF_u_* formula values and the experimental values are shown in Table 7 and Table 8. The deviations between the fitted *DIF_y_* modified Cowper–Symonds formula values and the average experimental values were below 3.6%, and the maximum deviation was below 3.8%. The deviations between the fitted *DIF_u_* modified Cowper–Symonds formula values and the average test results were below 5.1%, and the maximum deviation was below 5.4%. These small errors indicate that the modified *DIF_y_* and *DIF_u_* Cowper–Symonds formulas for HRB500E rebars spliced using sleeves are accurate.

Wang [17] et al. proposed the following Cowper–Symonds formula for *DIF_y_* of HRB500E rebars:

(9) $$DIF_{y}=\frac{f_{dy}}{f_{sy}}=1+{\left(\frac{\dot{\varepsilon }}{D_{3}}\right)}^{\frac{1}{q_{3}}}$$

where *D*_3_ = 264,713, *q*_3_ = 4.906.

and CEB Bulletin [26] proposed the following formula:

(10) $$DIF_{y}=1+\frac{m}{f_{y}}\ln\left(\frac{\dot{\varepsilon }}{{\dot{\varepsilon }}_{0}}\right)$$

where $\dot{\varepsilon_{0}}$ represents the quasi-static strain rate (0.0001/s), $\dot{\varepsilon }$ is the strain rate, *fy* stands for the yield strength of rebars, and parameter *m* is equal to 5.1. These two formulas will be used to analyze the effect of sleeved connections on rebar *DIF_y_*.

**Figure 8:**
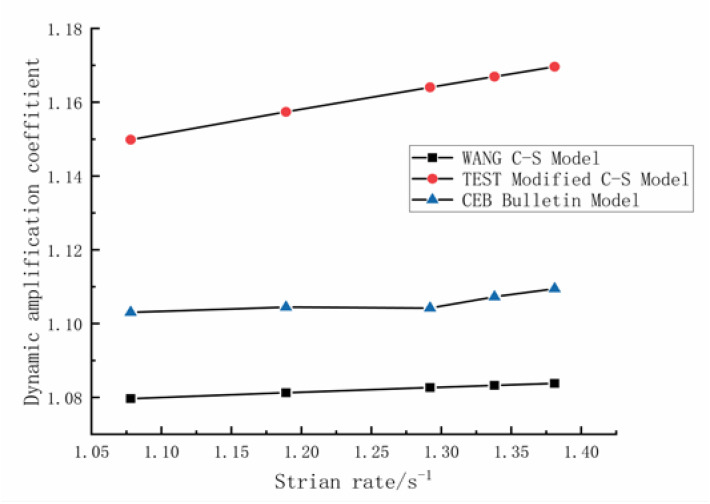
Comparison of *DIF_y_*.

A comparison of *DIF_y_* values calculated for the strain rates adopted in the test using Equations (9) and (10) as well as the modified Cowper–Symonds formula proposed in this paper is shown in Figure 8. It can be seen in Figure 8 that the *DIF_y_* values for the HRB500E rebars spliced with sleeves were larger than those of unspliced rebars. Denote generally by *ζ* the ratio of *DIF_y_* of rebars with sleeves to unspliced rebars, and specifically by *ζ_1_* the *DIF_y_* ratio between the Cowper–Symonds formula modified in this paper and the formula given by Wang et al., and by *ζ_2_* the *DIF_y_* ratio between the Cowper–Symonds formula modified in this paper and the CEB Bulletin formula. The trends in *ζ_1_* and *ζ_2_* with the strain rate are shown in Figure 8.

As seen in Figure 8, the *DIF_y_* ratios of rebars spliced using sleeves to unspliced rebars, *ζ_1_* and *ζ_2_*, increases with the increase in strain rate, demonstrating that the higher the strain rate is, the greater the influence of extrusion sleeves on the *DIF_y_* of rebars.

## 5. Conclusions

The dynamic tensile tests on the HRB500E rebars spliced with special impact-resistant extrusion sleeves at five different strain rates (1.079~1.395/s) were carried out using an electric-hydraulic tensile impact test machine with a maximum loading speed of 1500 mm/s and a maximum impact force of 2000 kN. The dynamic mechanical properties of rebars spliced with sleeves within the strain rate range adopted in the test were analyzed based on the experimental results, and the Cowper–Symonds formulas for *DIF_y_* and *DIF_u_* were modified. In addition, the *DIF_y_* values of rebars with sleeves and unspliced rebars were compared, and the influence of the novel extrusion sleeve on the strain rate effects of rebars was discussed. The main conclusions are as follows:

(1) At all strain rates tested, the specimens always failed due to the failure of the rebars themselves rather than the spliced connections. The average distance from the rebar fracture location to the joint satisfied the requirement that the rebar joints crack outside the joint length, which verified the reliability of the joint in the strain rate range of the impact (1.079~1.395/s).
(2) The relative total elongation at maximum force, *A_gt_*, of rebars spliced with sleeves decreased exponentially with the increase in strain rate, displaying clear strain-rate sensitivity. In the strain rate range likely to occur during impact, *A_gt_* of all specimens was above 5%, which complied with the technical requirement of the Hualong One reactor for the rebar mechanical splice joints (*A_gt_* was no less than 5%). However, when the strain rate exceeded 1.381/s, *A_gt_* of specimens was less than 7.5%, failing to meet the technical requirement for the mechanical rebar splicing of the French EPR nuclear reactors (*A_gt_* was greater than 7.5%).
(3) The deviations of *DIF_y_* (the yield strength of rebars with sleeves) and *DIF_u_* (the ultimate strength of rebars with sleeves) calculated by the modified Cowper–Symonds formulas and the average value of the test results were less than 3.6% and 5.4%, respectively. These errors were small, which indicated that the modified Cowper–Symonds formulas accurately reflect the strain rate effect on the strength of the rebars spliced with sleeves.
(4) The *DIF_y_* of rebars spliced using sleeves was larger than that of unspliced rebars, suggesting that the sleeves could improve the *DIF_y_* of rebars. The DIF ratios, *ζ*, of rebars with sleeves to unspliced rebars increased with the increase in strain rate, which indicated that the higher the strain rate is, the greater the influence of extrusion sleeves on the DIF of rebars.

## Figures and Tables

**Table 1:** Tensile strength requirements of steel bar joints.

Joint Grade	Class I	Class II	Class III
Tensile strength	*f*^0^*_mst_* ≥ *f_stk_* Broken in rebar or *f*^0^*_mst_* ≥ 1.10*f_stk_* Broken in joint	*f*^0^*_mst_* ≥ *f_stk_*	*f*^0^*_mst_* ≥ 1.25*f_yk_*

*f*^0^*_mst_*—Measured tensile strength of joint specimen; *f_stk_*—Standard value of tensile strength of reinforcement; *f_yk_* —Standard value of yield strength of reinforcement.

**Table 2:** Quasi static measured mechanical property indexes of HRB500E.

	*D*/mm	Yield Strength/MPa	Ultimate Tensile Strength/MPa	Total Elongation at Maximum Force/%
National standard requirements		≥500 MPa	≥630 MPa	≥9%
HRB500E	16	551.34	681.32	11.0
	20	561.15	689.47	10.2
	25	575.87	697.76	11.5
	32	570.43	747.72	10.3
	40	573.39	711.12	11.1

**Table 3:** Test design table.

Test Group	Total Length of Specimens/mm	Clamping Length/mm	Valid Length/mm	Strain Rate/s
Φ16	1250 ± 5	250	1000	1.395
Φ20				1.348
Φ25				1.298
Φ32				1.189
Φ40				1.079

**Table 4:** Analysis table of failure mode of test piece.

Test Group	Strain Rate//s	Failure Mode	Distance from Fracture to Joint/mm	Distance Requirement from Fracture to Joint/mm	Whether it Meets the Requirements
Φ16	1.395	Broken in the rebar	282	≥32	meet
Φ20	1.348		355	≥40	meet
Φ25	1.298		194	≥50	meet
Φ32	1.189		307	≥64	meet
Φ40	1.079		177	≥80	meet

**Table 7:** Deviation analysis table of DIFy fitting value and experimental value.

Strain Rate//s	Deviation of the DIFy Fitting Value from the Average Value of the Experimental Value	Maximum Deviation between DIFy Fitting Value and Experimental Value
1.395	1.8%	1.9%
1.348	0.1%	0.4%
1.298	3.2%	3.5%
1.189	1.9%	2.1%
1.079	3.6%	3.8%

**Table 8:** Deviation analysis table of DIFu fitting value and experimental value.

Strain Rate//s	Average Deviation between DIFu Fitting Value and Experimental Value	Maximum Deviation between DIFu Fitting Value and Experimental Value
1.395	0.8%	1.3%
1.348	2.7%	2.9%
1.298	3.7%	3.8%
1.189	1.4%	1.7%
1.079	5.1%	5.4%

## Data Availability

The dataset used in this study is included in the attachment.
